# Recent Progress in Deciphering the Etiopathogenesis of Primary Membranous Nephropathy

**DOI:** 10.1155/2017/1936372

**Published:** 2017-08-17

**Authors:** Andreas Kronbichler, Jun Oh, Björn Meijers, Gert Mayer, Jae Il Shin

**Affiliations:** ^1^Department of Internal Medicine IV (Nephrology and Hypertension), Medical University Innsbruck, Anichstraße 35, 6020 Innsbruck, Austria; ^2^Pediatric Nephrology, University Medical Center Hamburg-Eppendorf, Martinistraße 52, 20246 Hamburg, Germany; ^3^Department of Nephrology, UZ Leuven, Leuven, Belgium; ^4^Department of Immunology and Microbiology, KU Leuven, Leuven, Belgium; ^5^Department of Pediatrics, Yonsei University College of Medicine, Severance Children's Hospital, Seoul, Republic of Korea

## Abstract

Primary membranous nephropathy (MN) is the leading cause of nephrotic syndrome in adults. Discovery of several antibodies has contributed to an increased understanding of MN. Antibodies against the M-type phospholipase A2 receptor (PLA2R) are present in 50–100% with primary MN and are associated with a lower frequency of spontaneous remission. High levels are linked with a higher probability of treatment resistance, higher proteinuria, and impaired renal function, as well as a more rapid decline of kidney function during follow-up. Immunologic remission precedes reduction of proteinuria by months. Pretransplant evaluation of PLA2R antibodies is warranted to predict recurrence of disease following renal transplantation. Several risk alleles related to the PLA2R1 gene and within the HLA loci have been identified, whereas epitope spreading of PLA2R may predict treatment response. More recently, thrombospondin type 1 domain-containing 7A (THSD7A) antibodies have been discovered in primary MN. Several other rare antigens have been described, including antibodies against neutral endopeptidase as a cause of antenatal MN and circulating cationic bovine serum albumin as an antigen with implications in childhood MN. This review focuses on the progress with a special focus on diagnostic accuracy, predictive value, and treatment implications of the established and proposed antigens.

## 1. Introduction

Recent research in the field has unraveled important pathogenetic insights in the onset of primary membranous nephropathy (MN). Distinction between primary and secondary cases (mainly autoimmune disorders, infections, medications, and malignancy), characterized by a recognizable etiology, is pivotal. A clear difference from a histopathologic perspective is difficult, but absence of phospholipase A2 receptor (PLA2R) glomerular staining, preponderance of IgG1-3, and presence of extensive mesangial or subendothelial electron dense deposits may have a value in the diagnosis of secondary cases [1, 2]. While IgG1 is the predominant IgG subclass observed in secondary cases (60% of biopsies), IgG4 is predominantly found in subjects with primary MN (76% of biopsies) [3]. The exact role of the least represented IgG subclass is not completely understood, but it may exhibit distinct roles compared to IgG1 in allergy and tumor growth [4], and has attracted considerable interest due to increased incidence and prevalence of IgG4-related disease [5, 6].

Globally, MN represents the leading cause of nephrotic syndrome, with a reported annual incidence ranging from 0.2/100.000/year to 1.4/100.000/year and an overall estimate of 1.2/100.000/year [7]. A male predominance has been reported in most studies [7, 8], prognosis is related to renal function decline, and outcome tends to be better in females [8]. In general, patients present with a greater degree of proteinuria compared to those with focal segmental glomerulosclerosis (FSGS) and minimal change disease (MCD), but renal survival is assumed to be better compared to those having FSGS [9–11]. In line with FSGS [12], recurrence of disease after kidney transplantation takes place in approximately 35% of patients [13]. Recurrence is supposed to occur due to a yet-to-be-defined circulatory factor in FSGS, while progress in the understanding of MN has led to identification of circulatory factors leading to disease onset. While neonatal disease is associated with antibodies against neutral endopeptidase [14], M-type phospholipase A2 receptor antibody (PLA2R-Ab) [15], and thrombospondin type 1 domain-containing 7A (THSD7A) [16] autoantibodies are responsible for approximately 80% of primary MN cases. The aim of this review is to summarize recent findings in primary MN, focusing on implications of identified antibodies as well as their diagnostic accuracy and therapeutic impact.

## 2. M-Type Phospholipase A2 Receptor Autoantibodies and Glomerular Phospholipase A2 Receptor in Primary Membranous Nephropathy

PLA2R-Abs were first identified by Western-blotting and subsequent mass spectrometry in 26 out of 37 (70%) patients with primary MN. The 185-kD protein was absent in secondary MN as well as other proteinuric diseases (diabetic kidney disease, FSGS, or other autoimmune disorders). Moreover, samples reacting with the glycoprotein recognized recombinant PLA2R in vitro and PLA2R could be detected in human glomeruli. Glomerular deposits of patients with primary MN had a predominance of IgG4-subclass antibodies, and PLA2R colocalized with IgG4 in glomeruli [15]. A subsequent large multicenter study from Europe including 117 patients revealed presence of PLA2R-Ab in 74% and 72% using an immunofluorescence test (IFT) or ELISA, which increased to 76% when positivity in either test was considered as positive finding. The IgG4 subclass was present in 69% of all patients and correlated best with antibody positivity measured by IFT or ELISA [17]. Analysis of PLA2R staining in MN revealed positivity in 69% of 88 patients and was accompanied by PLA2R-Ab positivity in 60/61 patients (different time points of blood sampling). Enhanced PLA2R staining was exclusively found in primary MN and, of note, IgG4 staining was present in 95% of primary MN and in only 20% with secondary causes [18]. A study from the Netherlands investigated 18 samples during active disease and found a frequency of 77.8% PLA2R-related MN in their cohort [19]. In a prospective study by Hoxha and colleagues, PLA2R-Ab were measured in treatment-naïve patients. PLA2R-Ab were detected in 133/163 (81.6%) patients [20].

Several studies from Asia investigated presence of PLA2R-Ab and glomerular PLA2R staining. In a Korean study, 69/100 patients (69%) had detectable PLA2R-Ab as assessed by Western blot [21]. PLA2-related MN as detected either by IFT, by ELISA, or by glomerular PLA2R staining was reported in 94/114 (82.5%) in a study from India [22]. A study from China including consecutive patients with primary MN reported a frequency of 64.6% (53/82) [23]. In contrast to this low frequency of antibody positivity, another study from China found PLA2R-Ab in 49/60 patients (81.7%). When decreasing the dilution of samples for Western blot analysis, 10 of the remaining samples had low titers, albeit with a weaker strength compared to the other positive samples [24]. A larger study including 572 patients found detectable PLA2R-Ab in 68.5% (*n* = 392) as assessed by ELISA. Among the antibody negative cases (*n* = 180), glomerular staining was present in 70.6% (*n* = 127) [25]. Similar high frequency of PLA2R-MN (92.2%) was reported from another Chinese study analyzing 179 primary MN patients [26]. In Japanese patients, the prevalence of PLA2R-Ab may be lower compared to the rest of the world. A retrospective analysis of patients with 100 primary MN cases (after screening for clinical or laboratory signs of a secondary cause) revealed positivity in 53% of patients, with a higher prevalence observed in these subjects with nephrotic range proteinuria (61% versus 43%) [27]. Another study found PLA2R-Ab in 50% of their patients, whereas similar frequency of positive glomerular PLA2R staining could be detected in two cohorts (52.6 and 52.7%, resp.) [28, 29]. The respective studies reporting adult-onset primary MN are summarized in Table 1.

Analysis of PLA2R-Ab screening during remission showed a limited predictive value to identify PLA2R-related MN, with 8 out of 37 (22%) samples testing positive, whereas PLA2R was found in 22 of 37 (59%) corresponding biopsies [43]. PLA2R-Ab levels have been examined in childhood MN and were present to a lesser extent in a pediatric population, ranging from 2/34 (5.9%) to 10/22 (45.5%) patients reported from Japan and Northern America [44, 45]. In contrast, Kumar and coworkers reported PLA2-related MN in 15/18 (83.3%) of adolescents with primary MN [46].

In general, discrimination between primary and secondary cases is pivotal. In individual studies, a substantial heterogeneity has been reported. To better characterize PLA2R-Ab as a marker for primary MN, two meta-analyses were conducted. The more recent study by Dai and colleagues included 19 studies with 1160 patients. PLA2R-Ab positivity yielded a sensitivity of 0.68, a specificity of 0.97, a diagnostic odds ratio of 73.75, and an area under the curve of 0.82 when comparing primary and secondary MN. The respective values for glomerular PLA2R staining were 0.78, 0.91, 34.70, and 0.84 for the same parameters without heterogeneity [47].

The GEMRITUX trial comparing nonimmunosuppressive antiproteinuric treatment (NIAT) with rituximab against NIAT alone found PLA2R-Ab in three subjects who were initially found to be negative [38]. In line, “seroconversion” has been reported in a recent case series and a “kidney in the sink”-hypothesis has been generated, discussing the role of a PLA2R-Ab buffer capacity exhaustion of the kidney leading to antibody detection at a later stage [48]. In contrast to this assumption, a case report reported presence of PLA2R-Ab in a patient eight months ahead of MN presentation. PLA2R-Ab was measured during a thrombophilia work-up [49].

Taken together, the ideal time point of sample collection is at the time of kidney biopsy and ahead of treatment initiation, which may account for misclassification as PLA2R-Ab negative MN in some cases. Detection of serum PLA2R-Ab by diverse commercial measures is reliable, but cases of PLA2R-related MN may be missed by antibody detection only. Thus, staining for PLA2R antigen in the kidney should be considered to overcome this limitation. A preponderance of IgG4 is found in cases with PLA2R-related MN in serum and kidney specimens. Presence of PLA2R-Ab show a lower prevalence in Japan (around 50%) and in early-onset MN compared to other investigated cohorts (PLA2R-Ab positivity ranging from 65 to almost 100%). While PLA2R-Ab detection and predominant IgG4 presence may be considered a good measure to discriminate primary from secondary causes of MN in Caucasians, this is of lower validity in Chinese patients as shown by the recent meta-analyses.

### 2.1. Secondary Membranous Nephropathy and PLA2R Ab or PLA2R Staining Positivity

Initial studies have reported PLA2R-Ab positivity occurring in primary MN only. With the increasing body of evidence, secondary MN forms positive for either PLA2R-Ab or glomerular PLA2R staining have been identified.

In a cohort of patients with membranous lupus nephritis (LN), PLA2R-Abs were undetectable [30]. This is in line with Japanese studies reporting PLA2R-Ab positivity exclusively in patients with primary MN [27] or absence of either PLA2R-Ab or glomerular staining for PLA2R [28], whereas a low frequency (5.4%) could be observed in another analysis (one case HBV-associated and one malignancy-related) [29]. Positive PLA2R staining had a sensitivity of 75% and a specificity of 83% for primary MN in a large US study (85 primary and 80 secondary cases). Predominant secondary causes were hepatitis C (7/11, 64%), sarcoidosis (3/4, 75%), and malignancy (3/12, 25%), whereas positive staining was found to a lesser extent in patients with autoimmune diseases (1/46, 2%). Interestingly, all PLA2R positive secondary MN cases showed IgG4-predominant staining [31]. A French nationwide survey retrospectively analyzed kidney biopsies from patients with sarcoidosis and found PLA2R antigen in immune deposits in all patients. Serial serum samples were obtained in two patients and circulating PLA2R-Ab levels were in accordance with sarcoidosis activity and both, PLA2R-Ab and PLA2R, were predominantly related to the IgG4 subclass [50].

In a Chinese study, 84% of 102 tissue samples of patients with primary MN were PLA2R positive. Among secondary causes, one out of 38 patients with class V LN and 64% with hepatitis B virus-associated MN (HBV-MN) showed positive PLA2R staining [32]. Of 22 cases with secondary MN (HBV-MN, LN or both), 36.4% (8/22) had detectable PLA2R-Ab [23]. Analysis of 24 patients with psoriasis and MN revealed PLA2R-Ab and PLA2R positivity in 7 (29.2%) subjects. Among the patients with PLA2R-positivity, a majority showed IgG4 predominance on immunofluorescence (IF) [33]. Secondary causes of MN were accompanied by detectable PLA2R-Ab in a minority of patients in a Chinese study, including 1/20 positive cases with LN, 1/16 with HBV-MN, and 3/10 with malignancy-associated MN. In line with other observations, IgG4 was the predominant PLA2R-reactive subclass [24]. A Korean study found PLA2R-Ab in two out of nine patients with secondary MN (1 HBV and 1 malignancy-associated) and both showed predominant IgG4 immunostaining [21]. Positive PLA2R-staining was found in 2/26 (7.7%) of patients with HBV-MN and 2/2 (100%) presenting with malignancy. All patients with secondary causes and detectable glomerular PLA2R staining had predominant IgG4 deposition [26]. The respective findings are summarized in Table 2.

A preponderance of IgG4 staining on IF is assumed to be present in primary MN. As discussed by De Vriese and colleagues, the question arises whether some of these cases represent true secondary MN or rather PLA2R-related MN with coincident secondary diseases [51]. A higher frequency of secondary causes associated with PLA2R-Ab or PLA2R staining has been reported in Chinese studies, especially cases with hepatitis B-associated MN known to be endemic in China. Among the diseases with antibody positivity or positive staining, hepatitis B- or C-associated MN, malignancy-associated MN, and sarcoidosis emerged to be predominantly found.

### 2.2. Correlation of PLA2R-Ab Titers with Clinical Characteristics

In a study including 101 primary MN patients, baseline creatinine levels appeared to be higher in PLA2R-Ab positive patients, whereas baseline rate of proteinuria correlated with PLA2R-Ab levels. Spontaneous remission was recorded more often in patients being PLA2R-Ab negative [34]. Analysis of a Dutch cohort (*n* = 77) revealed a correlation of PLA2R-Ab titers with baseline proteinuria, serum creatinine, and baseline estimated glomerular filtration rate (eGFR) after adjustment for fractional IgG excretion (as representative of urinary loss of antibodies). Dividing patients into tertiles based on ELISA titers, renal failure tended to occur more frequently in patients belonging to the group in the highest tertile (PLA2R-Ab titer > 610 U/ml) and these patients had a significantly lower incidence of spontaneous remission compared to the lowest (PLA2R-Ab titer 41–175 U/ml) tertile (4 versus 38%, resp.). Accordingly, physicians were prompted to initiate immunosuppression more often in patients in the highest tertile compared to both other groups, whereas time to treatment response was significantly shorter in patients within the lowest tertile [17]. A single-center study from the same institution including 18 primary MN cases highlighted correlation of PLA2R-Ab with proteinuria, serum ß2-microglobulin (ß2m), urinary IgG excretion, urinary ß2m, and serum creatinine, whereas eGFR was negatively correlated [19]. Another large study including 118 PLA2R-Ab positive patients established different tertile values, with tertile 1 ranging from 20 to 86 U/ml, tertile 2 from 87 to 201 U/ml, and tertile 3 > 202 U/ml. Patients in tertile 3 were older, had a higher baseline systolic blood pressure, and tended to have more severe proteinuria. The proportion of patients reaching the predefined primary end point, namely, serum creatinine increase ≥ 25% and a creatinine value ≥ 1.3 mg/dl, was significantly higher in tertile 3 compared to tertile 1 (69% versus 25%). At the end of follow-up, proteinuria and PLA2R-Ab levels were higher in patients belonging to tertile 3 [35]. In a cross-sectional analysis, risk of progressive kidney function decline over a long observation period (up to 5 years) was increased in patients with higher PLA2R-Ab levels (high titer > 88 *μ*/ml versus low titer < 24 *μ*/ml) [36]. In the GEMRITUX study, a titer of < 275 U/ml was significantly associated with achieving the primary end point (complete or partial remission) [38]. A clear association between proteinuria and serum PLA2R-Ab levels and serum creatinine could be confirmed by a prospective study including 133 patients with detectable antibodies. PLA2R-Ab titers at baseline were significantly higher in those patients who did not experience remission during follow-up. Patients having lower PLA2R-Ab levels reached remission significantly faster compared to those with higher titers at baseline [20]. Another study from India observed a significantly higher proportion of complete remissions in the combined lower and intermediate range PLA2R-Ab tertile compared to those belonging to the highest tertile. Furthermore, resistant disease was common in patients with a baseline titer > 300 RU/ml (55.2%), whereas remission was frequently observed in those with a titer of < 100 RU/ml at baseline (68.2%). In general, detectable PLA2R-Abs were associated with treatment resistance compared to patients with no antibodies [22]. In a large study from China, detectable PLA2R-Abs (*n* = 392) were associated with a higher level of proteinuria and a lower level of eGFR. A weak correlation of antibody levels with proteinuria, serum albumin, and eGFR was shown. Moreover, patients with a positive PLA2R-Ab test and glomerular PLA2R staining were more likely not to achieve remission compared to those with undetectable antibodies and positive glomerular staining. In general, patients with a higher PLA2R-Ab or glomerular PLA2R rate had a higher proportion of failure to achieve remission. Combined positivity was a significant predictive value for no remission [25]. In line with several other reports, PLA2R-Ab positivity in Korean patients was associated with more severe proteinuria and lower serum albumin [21]. Proteinuria and number of patients having nephrotic range proteinuria were significantly higher in PLA2R-Ab positive cases in a Chinese study [23]. In a Japanese analysis including 19 patients with and without detectable PLA2R-Ab in each group, serum IgG was significantly lower in patients with antibodies, whereas proteinuria tended to be higher [28]. Respective associations/correlations have been highlighted in Table 3.

Taken together, based on the available data it is not possible to provide definite PLA2R-Ab cut-off, since different values have been reported in the respective studies. Nevertheless, in several studies, patients within the highest tertile had worse renal outcome and more frequently treatment resistance towards initiated immunosuppression. Notably, two reports have used purified protein to coat ELISA plates [17, 36], whereas the remainder used a commercially available kit [22, 35, 38]. High PLA2R-Ab titers (exceeding 250–300 IU/L) have been associated with more severe proteinuria, higher serum creatinine/lower eGFR, serum/urinary ß2M, and urinary IgG excretion.

### 2.3. PLA2R-Ab Presence and Decline Predict Treatment Response

PLA2R-Abs were present during clinical disease activity, whereas a decline or complete disappearance of PLA2R-Ab preceded resolution of proteinuria [15]. In a first multicenter retrospective study, the effects of rituximab on PLA2R-Ab levels were tested. 25 of 35 (71%) patients had detectable PLA2R-Ab and these antibodies disappeared or declined in 17 (68%) patients within 12 months following rituximab treatment, accompanied by a higher rate of complete or partial remission compared to those with a persistent antibody titer (59% and 88% versus 0% and 33% after 12 and 24 months). Changes in antibody titers preceded changes in proteinuria [40].

An observational study assigning patients either to oral cyclophosphamide or to mycophenolate mofetil and corticosteroids for 12 months evaluated PLA2R-Ab (*n* = 34/48) in retrospective. By 2 months of treatment, PLA2R-Ab levels declined from 428 U/ml to 24 U/ml. Antibody status at the end of treatment predicted 5-year outcome, with 58% (14/24) of patients with absent PLA2R-Ab achieving persistent remission compared to 0 out of 9 in those with persisting antibodies [41]. Combined treatment with rituximab and cyclosporine for 6 months of revealed PLA2R-Ab negativity in 77.8% after 3 months and in all at least once during follow-up, which was accompanied by remission. In two patients with relapse, reemergence of PLA2R-Ab was associated with re-occurrence of proteinuria [52]. A recent randomized controlled trial comparing NIAT and rituximab against NIAT alone found significantly more PLA2R-Ab negativity and decreasing titers in the NIAT-rituximab group. In the group with immunologic remission, the primary end point (complete or partial remission after 6 months) was achieved in 43% compared to 18% with persistent antibody positivity [38]. In 81 antibody-positive patients receiving a B-cell driven (375 mg/m^2^ once and a second infusion when more than five circulating B-cells/mm2 were detected after one week) or a fixed regimen (375 mg/m^2^ four times, one week apart), a lower PLA2R-Ab titer and full depletion after six months significantly predicted remission. In general, antibody decline preceded proteinuria and, particularly, a 50% reduction of PLA2R-Ab preceded equivalent proteinuria reduction by 10 months. Reappearance of PLA2R-Ab predicted disease relapse [37]. A similar pattern of PLA2R-Ab course was found in a Dutch study, showing a significant decline in the remission phase and a reappearance of PLA2R-Ab during relapse [19]. In a prospective German study, a decrease in PLA2R-Ab levels was accompanied by a steady increase in serum albumin. After three months of follow-up, a 45% decrease of PLA2R-Ab titers was paralleled by a 25% decrease in proteinuria in a cohort of 133 patients. The changes were even more pronounced in patients receiving immunosuppression, with a decline in PLA2R-Ab levels ranging from 69 to 81% and proteinuria by 38.8% [20]. In another analysis from India including 114 patients, a decline over 90% in PLA2R-Ab decline in PLA2R-Ab titers by 12 months was accompanied by remission in 85.4%, whereas a decline lower than 50% in the same period was associated with persistent nephrotic range proteinuria in 87.5%. A correlation between proteinuria and serum albumin with PLA2R-Ab titers at 6 and 12 months was found [22]. In a recent Chinese study comparing PLA2R-related MN (*n* = 91) with negative patients (*n* = 13), the total remission rate was significantly higher in patients with absent PLA2R-Ag staining [39].

A subanalysis of the prospective study conducted by Hoxha and colleagues analyzed the clinical outcome of included patients with nonnephrotic range proteinuria. During the follow-up period, significantly more patients with detectable PLA2R-Ab developed nephrotic range proteinuria (13 out of 16 patients) compared to those that tested negative (five out of 17). In accordance with this finding, immunosuppression was initiated more often in patients with PLA2R-Ab (13 versus 2 patients, resp.). At the end of follow-up, persistence of PLA2R-Ab was associated with no remission of proteinuria and a decline in renal function [42]. Findings have been highlighted in Table 4.

Taken together, higher baseline PLA2R-Ab titers are associated with worse renal outcome and resistance towards immunosuppressive therapy. In general, decline in antibody levels precede changes in proteinuria by months, irrespective of the immunosuppressive measure. Moreover, re-appearance of PLA2R-Ab is a strong predictor of disease relapse.

### 2.4. Recurrence of Disease after Transplantation and PLA2R-Ab

In a study by Quintana and colleagues, recurrence was observed in 7 of 21 (33.3%) patients. PLA2R-Abs were measured ahead of kidney transplantation and were positive in 85.7% and 35.7% of the recipients with and without recurrence, respectively. A correlation of PLA2R-Ab positivity at the time of graft biopsy and before transplantation with recurrence of primary MN was found by the authors. A PLA2R-Ab cut-off level of 45 U/ml could predict recurrence, with a sensitivity of 85.3% and a specificity of 85.1%. Presence of* HLA-DQA1* 05:01(05) and* HLA-DQB1* 02:01 was reported in 6 of 7 patients with recurrent MN and was associated with the highest pretransplantation PLA2R-Ab levels [53]. These findings were in part corroborated by another single-center study. Among the six patients (37.5%) with recurrence, a positive ELISA with a titer > 30 RU/ml provided a sensitivity and specificity of 83% and 100%, respectively. The risk alleles were found in 5 out of six patients (83%). Combination of both above-mentioned studies (*n* = 37) yielded a sensitivity of 85% and a specificity of 92% to predict recurrence with a PLA2R-Ab titer of > 29 U/ml [54]. Another study including 10 patients with primary MN reported an association of persistent IgG4 PLA2R-Ab and the respective antibody titer after six months with the recurrence of disease [55]. Moreover, persistence or reappearance of PLA2R-Ab after kidney transplantation was associated with increasing proteinuria and treatment-resistant recurrent MN in another case series [56]. Taken together, a PLA2R-Ab titer before renal transplantation > 30 or > 45 U/ml (depending on the respective publications), persistence, or reappearance during follow-up may predict recurrence of disease. This needs to be confirmed by further studies assessing patients in a prospective manner.

### 2.5. Immunodominant Epitope Region and Impact of Epitope Spreading on Outcome

Epitopes were identified by SPOT (peptide arrays on membrane) technology and detected by Western blot. Restricting analysis to the IgG4 subclass yielded lower background and stronger signals localized to seven consensus epitopes, which were in the extracellular domain of the PLA2R. Six of them were localized to the C-type lectin-like domains (CTLDs, one in CTLD1, one in CTLD2, two in CTLD6, and two in CTLD8), whereas one was located to the N-terminal cysteine-rich region (C-R) [57]. Further research aimed at deciphering the responsible epitope responsible for PLA2R-Ab binding. Fresquet et al. identified the major epitope within the N-terminus to CTLD3 (comprising a complex of CysR, FnII, and CTLD1–CTLD 3) of PLA2R which was recognized by 90% of human PLA2R-Ab and effectively competed for the binding with full-length PLA2R. Further experiments using mass spectrometry revealed a pivotal role of peptide 1 and peptide 2, representing the ß1 and ß3 strain from the ricin domain. Extension of peptide 1 towards peptide 2 is exerted by a 31-mer within the cysteine-rich (CysR) region, and competitive binding analyses revealed the essential role of 31-mer in the binding of PLA2R-Ab [58]. In line with the results described above, Kao and colleagues reported recognition of patient sera by a protein complex consisting of the CysR, fibronectin-like type II (FnII), and CTLD1 domains of PLA2R. Binding analysis comparing full-length PLA2R and the complex of CysR-FnII-CTLD1 indicated an equally efficacy in the recognition of PLA2R-Ab positive patient sera [59]. Since 10% of patients did not show binding to the described epitopes, it was assumed that a further epitope in the more C-terminal region may exist [60]. Further in depth analyses led to the identification of the three pivotal epitopes responsible for PLA2R-Ab binding. Seitz-Polski et al. described the loss of recognition in all tested patients (*n* = 50) after the deletion of the three epitopes, namely CysR, CTLD1 and CTLD7. Based on their findings, they set up ELISAs to specifically measure presence of antibodies directed against these domains. A total of 69 patients with presence of PLA2R were tested, and CysR as the dominant and most common epitope was recognized in 68 patients, whereas the CTLD1 and CTLD7 domains were recognized in 42 and 32 sera, respectively. Based on these findings, they classified patients according to antibody presence into a CysR (CysR antibodies only, *n* = 23), CysRC1 (CysR and CTLD1 only, *n* = 14) and a CysRC1C7 group (with presence of antibodies against all domains, *n* = 32). The CysR antibodies were significantly higher in the latter two groups compared to the CysR group. Patients in the CysR group were significantly younger and their baseline proteinuria was lower compared to the other two groups, while a proteinuria over 5 g/g creatinine was most frequently found in the CysRC1C7 group. Furthermore, a progressive increase of proteinuria was accompanied by a progressive increase in CTLD7. Assessment of outcome revealed significantly more spontaneous remissions in the CysR group, whereas the occurrence of end stage renal increase was limited to the CysRC1 and CysRC1C7 groups and proteinuria increased from CysR to CysRC1C7. In accordance, the proportion of patients with a 30% increase in serum creatinine was higher in the CysRC1 and CysRC1C7 groups. Multivariate analysis retained CysRC1, CysRC1C7 and high titers of PLA2R-Ab as independent risk factors for poor renal prognosis. Serial samples were available for a subset of patients and an epitope switch was observed in a minority, associated with a change in clinical presentation (i.e., one patients switched from the CysRC1C7 to the CysR group and entered remission following initiation of immunosuppression). ELISA tests were set up for the detection of IgG4 antibodies only. Analysis of other subclasses highlighted that anti-CTLD7 antibodies were exclusively restricted to the IgG4 subclass [61].

The identification of the immunodominant epitope regions of PLA2R have further provided insight into pathogenesis. Moreover, the CysRC1, CysRC1C7 and higher titers of circulating PLA2R-Ab may be considered as independent risk factors for poor prognosis. Moreover, epitope spreading has been observed, indicating changes in both directions (high risk phenotype to the CysR group and vice versa). It is tempting to speculate that specific developments (i.e., commercially available ELISA kits) will further help to facilitate identification of patients bearing a high risk of treatment resistance or progressing to end stage renal failure.

### 2.6. Identification of Susceptible Loci Associated with Primary Membranous Nephropathy

A genome-wide association study (GWAS) was employed to identify risk alleles responsible for the onset of primary MN. Three cohorts (with Dutch, French and British patients) were analyzed and independently displayed a significant association with* HLA-DQA1* on chromosome 6p21. In a joint analysis, comprising 556 patients with primary MN and 2338 controls, the authors identified the most significant association within* HLA-DQA1* and* PLA2R1* on chromosome 2q24 [62]. A subsequent study from Spain including 89 primary MN patients and 286 controls confirmed genetic associations with* HLA-DQA1* and* PLA2R1*. Interestingly, combination of the susceptibility genotypes (A/A and A/G for* HLA-DQA1* and A/A for* PLA2R1*) significantly predicted response to treatment, which became even more apparent after adjustment for baseline proteinuria. Moreover, carriers of the A/A or A/G genotype for* HLA-DQA1* had a longer mean doubling of serum creatinine-free survival [63]. A study from Germany observed an association between classical HLA alleles (*DRB1*^*∗*^0301-*DQA1*^*∗*^0501-*DQB*^*∗*^0201 haplotype) and primary MN. Whereas* PLA2R1* was exclusively identified in primary MN, the* HLA-DQA1* risk allele was associated with LN, diabetic nephropathy and FSGS as well [64]. In a cross-sectional study from the UK,* HLA-DQA1*^*∗*^05:01 and* HLA-DQB1*^*∗*^02.01 were associated with higher PLA2R-Ab levels [36]. In a large monocentric study, analysis of the risk alleles revealed that homozygous carriage of the* PLA2R1* A allele was associated with a 2-fold higher PLA2R-Ab level compared to A/G and G/G carriers [37].

Similar results were reported in non-Caucasian patients with primary MN. A study from India compared 114 cases and 95 healthy controls and observed differences in the* PLA2R1* and* HLA-DQA1* SNPs which became more evident when studying PLA2R-related cases only [22]. In a large Northern American analysis,* PLA2R1* SNPs were associated with PLA2R-related MN in Caucasians only. A strong epistasis between* PLA2R1* and* HLA-DQA1* was found [65]. In African Americans, 120 PLA2R-related MN biopsies were screened for the presence of* APOL1* risk alleles, identifying 46 cases with 0, 51 cases with 1 and 23 cases with 2* APOL1* risk alleles. Presence of two risk alleles was associated with histologic features implicated in worse renal outcome [66].

Analysis of 1112 Chinese patients and 1020 healthy controls revealed a strong association between three SNPs within* PLA2R1* and one SNP within* HLA-DQA1* and a potentiation when risk alleles were combined [67]. Restricting analysis to the MHC region in PLA2R-positive primary MN,* HLA-DRB1*^*∗*^15:01 and* HLA-DRB3*^*∗*^02:02 were strongly associated. Whereas* HLA-DRB3*^*∗*^02:02 was found in a comparable number of PLA2R-negative MN,* HLA-DRB1*^*∗*^15:01 was found in 81.8% of patients with PLA2R-related MN compared to 16% of PLA2R-negative MN and 21% of the healthy controls. A replication cohort confirmed the findings of both HLA loci. Interestingly, in a joint analysis, 387 patients (98.7%) carried at least one of the proposed HLA risk alleles [68]. Another Chinese study included 261 cases and 599 healthy controls.* HLA-DRB1*^*∗*^15:01 and* HLA-DRB1*^*∗*^03:01 were independently associated with primary MN. PLA2R-Ab positivity was associated with the carriage of one of the risk alleles, whereas association remained at a lower degree for* DRB1*^*∗*^15:01, while* DRB1*^*∗*^03:01 lost its effect [69]. In a Japanese cohort comprising 183 patients with primary MN and 811 healthy controls, several tested* PLA2R1* SNPs were associated. Moreover, the association of* HLA-DRB1*^*∗*^15:01 could also be observed, whereas* HLA-DQB1*^*∗*^06:02 emerged as a second independent risk allele [70]. To identify rare genetic variants in* PLA2R1*, all 30 exons of* PLA2R1* were sequenced. Although the general association between* PLA2R1* and primary MN could be confirmed, no evidence was provided that rare variants within the coding region cause the proposed association between primary MN and the* PLA2R1* gene [71].

Despite progress in the understanding of genetics related to primary MN, there is a lack of heritability. One report from 1984 highlighted occurrence of MN in three pairs of brothers, with one pair being monozygotic (HLA-identical) twins [72]. In general, the presence of* HLA-DQA1* and* PLA2R1 *risk alleles confers a significant association with primary MN and the combination of risk phenotypes potentiates the risk to develop MN in most ethnicities. Particularly, MHC class II molecules play a pivotal role in MN. In Chinese patients with primary MN, carriage of* HLA-DRB1*^*∗*^15:01 confers a significant risk to develop PLA2R-related MN, whereas* HLA-DRB1*^*∗*^03:01 or* HLA-DRB*^*∗*^06:02 may carry a risk to develop either PLA2R-related or unrelated MN.

## 3. Thrombospondin Type 1 Domain-Containing 7A in (Primary) Membranous Nephropathy

Since about 20–30% of cases with primary MN may exhibit other antigens, a similar approach as with the discovery of PLA2R-Ab was chosen to identify further antigens. Thrombospondin type 1 domain-containing 7A (THSD7A) was identified by Western-blotting and subsequent mass spectrometry and has a molecular size of approximately 250 kDa. Among patients with PLA2R-Ab negative primary MN, THSD7A was found in 6 out of 44 patients in a European and in 9 out of 110 patients in a US cohort. In accordance with PLA2R-Ab, IgG4 was the predominant IgG subclass of anti-THSD7A, and colocalization of IgG4 and THSD7A was observed in renal tissue specimens. Staining for nephrin colocalized with staining for THSD7A, suggesting expression of THSD7A in proximity to the podocyte foot processes. Interestingly, anti-THSD7A antibodies were more frequently observed in women (65% versus 27%) [16]. A single-center study from Japan including consecutive patients with MN found THSD7A-related MN in five out of 55 patients (9.1%). Again, total numbers were low, but the authors found that patients with THSD7A-related MN were significantly younger compared to PLA2R-related MN and a female predominance (three out of five), which was in contrast to patients being PLA2R-positive [29]. Lower prevalence was reported in a Chinese cohort (three patients with primary MN), and additionally two patients had secondary causes (one tumor-associated and one hepatitis B-associated, overall 3.7%). Importantly, IgG4 staining was positive in primary and secondary causes [73]. Analysis of consecutive biopsies from the Boston cohort revealed a positive staining for THSD7A in 7/258 (3%) patients, whereas two cases presented with dual (PLA2R and THSD7A) positivity (1%) [74]. Concomitant occurrence of THSD7A-related MN and mixed adenoneuroendocrine carcinoma of the gallbladder with positive staining in the kidney, the gallbladder and an affected lymph node highlighted a potential link between THSD7A-positivity and malignancy. Screening of 1009 patients with MN revealed further 25 patients with positive anti-THSD7A antibodies. Of these, seven had a malignancy [75]. Further analysis of consecutive MN patients revealed a prevalence of 2.6% out of 345 patients, with a majority being female. The association between THSD7A-related MN and malignant disease exceeded the number of PLA2R-related MN and malignancy. After inclusion of the retrospective cohorts and analysis of samples derived from 1276 MN patients, 8 out of 40 patients with detectable anti-THSD7A antibodies presented with malignancy and the median time from diagnosis of MN and development or detection of malignancy was 3 months. Again, a female predominance was reported by the authors. Achievement of complete remission was accompanied by undetectable anti-THSD7A antibody titers, whereas a majority of those with partial remission and one patient with no remission remained positive [76]. In contrast to PLA2R1, THSD7A is expressed on both murine and human podocytes, and administration of human anti-THSD7A antibodies bind to murine THSD7A, leading to proteinuria and a histopathology resembling MN [77]. This finding confirmed the assumption that antigen-antibody interaction is pivotal in the onset of MN.

Taken together, anti-THSD7A antibodies are a novel antigen which is more frequently present in Japanese patients with primary MN. In particular, research needs to decipher its association with malignancy and intensive screening may be warranted in positive patients, since the number of observed malignant diseases may be underestimated by the current reports due to a short follow-up period.

## 4. Rare Forms of Childhood Membranous Nephropathy and Potential Ways to the Detection of Novel Antigens

MN is a common cause of nephrotic syndrome in adults; it is rare, however in children [78, 79]. Since PLA2R and THSD7A do not seem to play any major role in infants and young children, other auto- or allo-antigens must looked for, after secondary causes have been excluded thoroughly, particularly malignancy. Recently, rare causes of MN were identified in children, such as antibodies to neutral endopetidase (NEP).

A case of antenatal MN with a severe, unusual histopathological presentation was further investigated, since the transplacental passage of nephritogenic antibodies was suspected to be causative. Sequential serum samples from the mother (before and after delivery) and serum of the infant after birth showed reactivity with glomerular capillary walls and the brush border. A 90 kDa antigen was found, and further experiments identified NEP as the antigenic fraction. Kidney biopsy of the infant and of rabbits after incubation with maternal IgG revealed colocalization of IgG antibodies and NEP in subepithelial immune deposits. The mother's granulocytes exhibited a lack of NEP expression, thus an alloimmunization process was discussed as responsible pathogenetic step [80]. Truncating mutations in the metallomembrane endopeptidase* (MME)* gene were found to be causative of alloimmunization during pregnancy, leading to the production of anti-NEP antibodies. Whereas infants of four mothers with mutations had oligoanuria, nephrotic syndrome or renal failure, all children of one mother had no overt renal disease. Analysis of anti-NEP antibodies revealed an approximately 10-fold lower titer for IgG as compared to those of the remaining mothers. Rapid improvement of kidney failure and nephrotic syndrome was observed in affected infants [14]. Two mothers were followed during their second pregnancy. One mother developed a marked increase in anti-NEP IgG1 and IgG4 antibodies. Severe disease occurred despite treatment strategies aiming at reducing its levels. The other mother produced anti-NEP IgG4 antibodies exclusively and delivery of a healthy newborn was possible [81]. More recently, two additional mothers (from Germany and Italy) bearing a truncating deletion in the* MME* gene were identified. The German mother produced predominantly anti-NEP IgG4 antibodies, whereas the Italian mother mainly produced complement-fixing anti-NEP IgG1 antibodies, the latter able to inhibit NEP enzymatic activity. Whereas the German child had rapid regression of all symptoms, the Italian child required treatment with intermittent peritoneal dialysis and an impaired renal function at the time of last follow-up was reported. These findings highlight that complement-fixing anti-NEP IgG1 antibodies are necessary to develop a severe disease phenotype [82].

Various reports have already indicated a role for allergens, including dietary allergens in the pathogenesis of renal glomerular diseases. Early studies demonstrated that some patients develop relapses after cow's milk exposure and elimination of milk from the diet could prevent relapses of lipoid nephrosis [83]. This has been explained by a dysregulation of the IL-13 system [84], leading to overproduction of IgE-specific antibodies of food compounds, including cow's milk and theoretically also bovine serum albumin (BSA). Anti-BSA antibodies, commonly prevalent in the general population, have been detected in high levels in 4 of 5 consecutive children (age < 5 years) and 7 of 41 consecutive adults with MN. Elevated levels of circulating BSA were also present in those children with high levels of anti-BSA antibodies. As shown in various animal models, the cationic form of BSA is able to induce MN. Subepithelial deposits of BSA were detected in children with both, high levels of circulating cationic BSA and antibodies, and in the absence of PLA2R [85].

Gel electrophoresis identified a few proteins which were repetitively recognized by MN sera. Among these, antibodies against aldose reductase (AR), manganese superoxide dismutase (SOD2) were elevated in MN compared to FGSG and normal controls. Analysis of kidney specimen revealed marked expression of AR and SOD2 in glomeruli of patients with MN, and colocalization of both, AR and SOD2, with IgG4 and the terminal complement complex [86]. A proteomic approach found three immune proteins in glomeruli of patients with MN, namely *α*-enolase (*α*ENO), elongation factor 2 and glycyl aminoacyl-tRNA synthetase. One of the factors, *α*ENO, fulfilled the criteria and was considered as autoantigen, colocalized with IgG4 in deposits, and was found to be elevated in 25% of 131 MN patients [87]. Moreover, PLA2R Ab levels correlated with antibodies against AR, SOD2 and *α*ENO [88]. Recently, Kimura found no difference in the frequency of *α*ENO antibodies between primary and secondary MN, although IgG4 antibodies were exclusively found in primary MN [89].

Taken together, rare antigens have been observed in antenatal or early-onset membranous nephropathy. In cases with anti-NEP antibodies, preponderance of IgG4 antibodies was associated with a good prognosis, whereas a predominance of IgG1 was accompanied by a severe disease phenotype. Moreover, a role for the cationic form of BSA to cause membranous nephropathy in children has been described and circulating antibodies have been observed. Rarer antigens have been discovered by gel electrophoresis. One can speculate that more antigens will be discovered in near future.

## 5. Conclusions and Outlook

The aim of the present review was to summarize recent literature related to the immunology of primary MN. While PLA2R-Ab represents the leading circulatory antibody in primary MN with a presence of 70–80%, the role of antibodies to THSD7A needs to be better defined, since malignancy-associated THSD7A-related MN has recently been reported to occur in a significant proportion of patients. Other antibodies leading to secondary forms of MN have not been described so far, and there is still room for further antibody discovery. Moreover, primary MN has a clear genetic basis, but reports related to genetic heritability are widely lacking. In addition, novel ELISA kits detecting different epitopes of PLA2R-Ab may pave the way to distinguish PLA2R-associated MN patients with a predictable poor and favorable outcome. Rare antibodies have been identified in childhood or antenatal MN. With this increasing research in mind, it is likely that we will learn more about the etiopathogenesis of primary and secondary MN in near future.

## Figures and Tables

**Table 1:** A summary of studies investigating the prevalence of either phospholipase A2 receptor antibodies, glomerular phospholipase A2 receptor staining, or both in primary membranous nephropathy. ELISA (Enzyme-Linked Immunosorbent Assay), IFT (Indirect Immunofluorescence Test), PLA2R (phospholipase a2 receptor), PLA2R-Ab (phospholipase a2 receptor antibody), WB (Western blot). ^*∗*^Dilution of serum samples was decreased to 1 : 10, and exposure time was increased to 10 minutes.

Reference	Country	Prevalence (PLA2R-Ab)	Detection method	Prevalence (PLA2R staining)	Predominant IgG subclass
[15]	USA	26/37 (70%)	WB		

[17]	Netherlands, France, UK	87/117 (74%), 84/117 (72%)	IFT, ELISA		IgG4 (81/87, serum)

[18]	Germany	60/88 (68%)	IFT	61/88 (69%)	IgG4 (58/61, biopsy)

[19]	Netherlands	14/18 (78%)	WB		IgG4 (14/14, serum)

[20]	Germany	133/163 (82%)	IFT, ELISA		

[21]	Korea	69/100 (69%)	WB		

[22]	India	75/114 (65%), 76/114 (67%)	IFT, ELISA	86/114 (75%)	

[23]	China	53/82 (65%) 51/82 (62%)	IFT, ELISA		

[24]	China	49/60 (82%) 59/60 (98%)^*∗*^	WB		

[25]	China	392/572 (69%)	ELISA	514/572 (90%)	IgG4 (97.7% (PLA2R-Ab/R +/+), 98.4% (PLA2R-Ab/R −/+))

[26]	China			165/179 (92%)	IgG4 (167/179, 93%)

[27]	Japan	53/100 (53%)	WB		

[28]	Japan	19/38 (50%) 19/38 (50%)	IFT, ELISA	20/38 (53%)	IgG4 (20/20, biopsy)

[29]	Japan			29/55 (53%)	IgG4 (28/29, biopsy)

**Table 2:** Secondary causes of membranous nephropathy and the prevalence of either PLA2R-Ab or positive glomerular PLA2R staining are summarized. AID (autoimmune diseases), ELISA (Enzyme-Linked Immunosorbent Assay), IgG4-RD (IgG4-related disease), IFT (Indirect Immunofluorescence Test), LN (lupus nephritis), PLA2R (Phospholipase A2 Receptor), PLA2R-Ab (phospholipase A2 receptor antibody), and WB (Western blot). ^*∗*^Notably, 25/39 (64%) of patients with hepatitis B-associated MN had detectable PLA2R staining, whereas PLA2R positivity was found in 1/38 (2.6%) of lupus nephritis cases. ^*∗*1^By decreasing the dilution, 2 additional cases of hepatitis B-associated MN were considered positive.

Reference	Country	Prevalence (PLA2R-Ab)	Detection method	Prevalence (PLA2R staining)	Predominant IgG subclass	Entities
[15]	USA	0/8 (0%)	WB			LN, hepatitis B
[30]	Sweden	0/25 (0%)	IFT			LN
[27]	Japan	0/31 (0%)	WB			AID, cancer, drugs, hepatitis B, others
[28]	Japan	0/21 (0%)	IFT, ELISA			LN, cancer, hepatitis B
[29]	Japan			2/37 (5%)	IgG4 (2/2, biopsy)	AID, cancer, drugs, hepatitis B, hepatitis C, thyroiditis
[31]	USA			14/80 (18%)	IgG4 (6/6, biopsy)	AID, cancer, hepatitis B, hepatitis C, HIV, syphilis
[32]	China			26/77 (34%)^*∗*^		LN, hepatitis B
[23]	China	8/22 (36%)	IFT, ELISA			LN, hepatitis B
[33]	China	7/24 (29%)	IFT	7/24 (29%)	IgG4 (5/6, biopsy)	psoriasis
[24]	China	5/46 (11%) 7/46 (15%)^*∗*1^	WB		IgG4	LN, cancer, hepatitis B
[21]	Korea	2/9 (22%)	WB			LN, cancer, hepatitis B, hepatitis C
[26]	China			2/69 (3%)	IgG4 (2/2, biopsy)	LN, cancer, hepatitis B, IgG4-RD

**Table 3:** Baseline characteristics and presence of PLA2R-Ab with respective associations and correlations with outcome and variables are highlighted. ß2m (ß2-microglobulin), eGFR (estimated glomerular filtration rate), PLA2R-Ab (phospholipase A2R-antibody). ^*∗*^Analysis of different variables after adjustment of the respective antibody titers for fractional IgG excretion.

Reference	Country	Number of patients	Correlations/associations
[34]	Netherlands	101	Baseline creatinine (*p* = 0.03) Antibody levels and proteinuria (*p* = 0.017) Spontaneous remission in seronegative patients (*p* = 0.038)

[17]	Netherlands, France	77/79	Antibody levels and proteinuria (*p* < 0.01)^*∗*^ Antibody levels and serum creatinine (*p* < 0.01) and eGFR (*p* < 0.01)^*∗*^ Spontaneous remission more frequently in patients within the lowest PLA2R-Ab tertile (*p* < 0.01) Highest tertile more often needed immunosuppression, and higher levels were associated with longer time to remission (*p* < 0.01)

[19]	Netherlands	18	Antibody levels positively correlated with proteinuria (*p* < 0.01), serum ß2m (*p* < 0.01), urinary IgG excretion (*p* = 0.03), urinary ß2m excretion (*p* < 0.01), and serum creatinine (*p* = 0.03) and negatively correlated with eGFR (*p* = 0.049)

[35]	Germany	118	Patients in the highest tertile were older (*p* < 0.01), had lower serum albumin (*p* = 0.04), and higher systolic blood pressure (*p* < 0.01) Antibody level (IgG) is associated with outcome (serum creatinine increase ≥ 25% or creatinine level ≥ 1.3 mg/dl) Patients in tertile 3 reached the outcome parameters more frequently (*p* = 0.03) and significantly faster Proteinuria was higher at the time of last follow-up in patients within tertile 3 (*p* = 0.03)

[36]	UK	90	High PLA2R-Ab levels associated with an increased risk of progression (*p* < 0.001)

[37]	Italy	81	Patients in the lowest tertile antibody group had lower proteinuria compared to both other groups

[38]	France	75	Lower PLA2R-Ab levels associated with achievement of the primary end point (complete or partial remission)

[20]	Germany	133	PLA2R-Ab levels correlated with proteinuria and serum creatinine Lower PLA2R-Ab titers were associated with a faster time to remission and remission was achieved more frequently

[22]	India	114	PLA2R-Ab positivity was associated with treatment resistance (*p* = 0.02) Higher levels were associated with resistant disease (55%); low titers were associated with remission (68%)

[25]	China	392	PLA2R-Ab positivity was associated with a higher level of proteinuria (*p* < 0.001) and a lower level of eGFR (*p* < 0.01) Antibody levels correlated with proteinuria (*p* < 0.01), serum albumin (*p* < 0.001), and eGFR (*p* < 0.001) Patients with glomerular positivity (undetectable antibodies) were more likely to achieve complete remission (*p* < 0.001)

[21]	Korea	69	PLA2R-Ab positivity was associated with lower serum albumin (*p* = 0.004) and higher levels of proteinuria (*p* = 0.003) and nephrotic range proteinuria was more frequently observed (*p* < 0.001)

[23]	China	53	PLA2R-Ab positivity was associated with higher proteinuria (*p* = 0.008) and nephrotic range proteinuria (*p* = 0.036)

[28]	Japan	19	Serum IgG levels were significantly lower in PLA2R-Ab positive patients Serum albumin correlated inversely with serum PLA2R-Ab titers

**Table 4:** Effects of treatment on PLA2R-Ab and variables of interest have been highlighted. The study by Wang et al. [39] included nonnephrotic patients only and all patients received nonimmunosuppressive treatment with inhibition of the renin angiotensin aldosterone system.

Reference	Country	Number of patients	Treatment	Effect of treatment
[40]	USA	25	Rituximab	17/25 had undetectable Ab levels within 12 months 59/88% attained remission by 12 and 24 months (compared to 0/33% in those with persistent PLA2R-Ab)

[41]	Netherlands	33	MMF, *n* = 15; CYC, *n* = 18	16/18 became Ab negative during 12 months of CYC versus 8/15 in the MMF group (*p* = 0.02) In general, remission was observed in 22/24 negative patients versus 3/9 positive patients at 12 months Antibody status at the end of therapy predicted long-term outcome (persistent remission and alternative outcome measures)

[38]	France	55	Rituximab, *n* = 27; Placebo, *n* = 28	PLA2R-Ab negativity: 14/25 and 13/26 at months 3 and 6 in the rituximab group; 1/23 and 3/25 at months 3 and 6 in the placebo group In the rituximab group, antibody depletion at month 3, 6 patients (43%) were associated with the primary end point (complete/partial remission), with 2/11 patients (18%) without antibody depletion

[37]	Italy	81	Rituximab	Low titer of PLA2R-Ab predicted achievement of primary end point (complete or partial remission, *p* = 0.001) PLA2R-Ab promptly decreased and preceded a comparable proteinuria response by approximately 2 years Depletion of PLA2R-Ab after 6 months predicted primary end point Reappearance of antibodies was associated with relapse (*p* < 0.001)

[19]	Netherlands	18	MMF or CYC	PLA2R-Ab decreased significantly during remission and increased again during relapse (*p* < 0.01)

[20]	Germany	133	CNI, alkylating agents or rituximab	Decrease in proteinuria was accompanied by an increase in serum albumin (*p* < 0.001) At 3 months, proteinuria fell by 25%, whereas PLA2R-Ab levels decreased by 45% PLA2R-Ab levels as a risk factor for not achieving remission

[22]	India	76	CNI or CYC	>90% reduction in PLA2R-Ab titer at 12 months from baseline, accompanied by remission in 85% (<50% Ab reduction, 88% had persistent nephrotic range proteinuria) Proteinuria (*p* > 0.0001, each) and serum albumin (*p* = 0.001 and *p* < 0.0001) correlated with PLA2R-Ab titer at 6 and 12 months A greater proportion of subjects in the first two tertiles had complete remission compared with patients in the highest tertile (*p* = 0.04)

[42]	Germany	16		In initially nonnephrotic patients, nephrotic range proteinuria developed more often in PLA2R-Ab positive patients (*p* < 0.005) Significantly more PLA2R-Ab positive patients received immunosuppressive therapy (*p* < 0.001)
